# Acute Hydrocephalas

**Published:** 1840

**Authors:** 


### Acute Hydrocephalus. By *David D. Davis*, M.D. M.R.S.L. Taylor and Walton, 1840. 8vo. pp. 309.

Dr. Davis thus describes the objects he has in view in publishing this work. "The first is to prove, that acute hydrocephalus is an inflammatory disease; and the second, is to establish the fact that it is curable equally, and by the same means with other diseases of inflammation." Of the first part of the book our analysis will be brief—the fact announced in it being, we believe, already well known to the profession: we will dwell rather more fully upon the treatment recommended by the author, not that this is however the novelty he seems to consider it, but, because any recommendations, from a physician in extensive practice, in reference to a disease so often fatal, cannot be too widely or too fully diffused.

### I. The Symptoms and Diagnosis.

It cannot be necessary that we should minutely detail the symptoms of a malady, so well known and so frequently described, as acute hydrocephalus. The author has presented his readers with a very vivid and faithful picture of the disease, and we will content ourselves with transcribing some of the signs he considers especially pathognomic or diagnostic. He divides the disease into three stages; viz.—the *formative* or premonitory; the stage of *active inflammation;* and, the period of *collapse.*

1. *Formative Stage.*—This, (the stage of turgescence of Golis,) he thinks especially denoted by the following signs;—slight giddiness and confusion upon moving the head; aching pains of the extremities, and at the back of the neck; disturbed sleep; loss of appetite, often attended by vomiting; scanty evacuation by stool and urine—an altered complexion and general appearance—a stumbling gait—irritable and altered temper and disposition—morbid acuteness of the external senses—irregular, but not quick pulse—harsh skin, and loss of strength.

2. *Inflammatory Stage.*—In some subjects the formative stage is very short, and the inflammatory stage is at once developed, with great fever and convulsion; and this is especially the case in vigorous children. Generally, however, the second stage approaches gradually, after the expiration of several days. The head then becomes very painful and very hot; a distressing sense of pressure over the eyes is present, while they are more and more sensible to light: the countenance becomes yet more altered and the complexion pale; the vomitings are frequent, especially when the body is moved, and a characteristic fætor of the breath is present. A dull pain in the region of the stomach and liver, great subsidence of the abdomen, obstinate constipation, rapid emaciation with accompanying debility, are often prominent symptoms; while, with these, the pulse is slow, intermitting or irregular, and the skin more and more flaccid: the little patient frequently carries its hand to the seat of pain.

3. *Stage of Collapse.*—Of this Dr. Davis considers the following among the most prominent symptoms: a marked dullness of the hitherto preternaturally excited senses; an oblique position in bed; involuntary movements of the extremities, and general restlessness; picking of the ears, mouth and nostrils, the hands being however directed in a trembling and uncertain manner; disordered vision and a convulsive movement of the eye-lids; the urine scanty, deep coloured and furnishing a white heavy sediment—the pulse weak and soft, but still irregular—much sighing—very offensive breath—sudden and loud screaming, and grinding of the teeth. After these symptoms have continued from four to seven days an illusory calm often occurs, during which, the patient temporarily recovers his senses and natural disposition. This, after probably exciting the hopes of the parents, and of the attendant if inexperienced, soon passes away, and the child gradually sinks. The fatal termination is often accompanied by hectic fever, convulsions, loss of vision, or palsy.

In reference to *diagnosis*, the author is not sufficiently minute; he contents himself with an accurate abstract of the principal symptoms, and says, that by comparing these with the symptoms of other febrile diseases, the distinctions between them will be easily found. This is all very well in books, wherein we distinguish diseases from each other with great facility; but, at the bed-side, we feel too often the difficulty of the matter, and would be glad enough to avail ourselves of the numerous hints large practical experience ought to furnish us with. There are few practitioners but have been embarrassed some time or another, in distinguishing between hydrocephalus and infantile remittent fever. Again, we are surprised that Dr. Davis, whose practice lies so much among young children, should have passed over, in utter silence, the cases adverted to by Abercrombie, Gooch and Marshall Hall, and termed by the latter hydrencephaloid disease: cases, which arising from opposite causes, and requiring entirely opposite treatment, yet often bear a wonderful resemblance to true hydrocephalus.

### II. PREDISPOSING CAUSES.

This is a good chapter. Of all the predisposing causes, Dr. Davis thinks *difficult dentition* to be the most frequent. During this process, the vascular tissues of the brain become overcharged, and more or less drowsiness prevails: this is often followed by pain and heat of the head, and a marked change in the disposition of the child; its eye grows dull, and it appetite is diminished. If the infant be not relieved, by the judicious interference of the medical man, hydrocephalus will probably follow; for, in nine out of ten cases of the disease, occurring in children during the first three years of life, it originates in this manner.

The practice of feeding infants upon *spoon diet*, is also a powerfully predisposing cause of diseases of the head. The child will often, on account of the palatable manner in which it is prepared, take this description of food with avidity, and may even seem to thrive upon it very well for a while; but at no very distant time, a week or two, or a month or two, symptoms of deranged digestion manifest themselves, and various organs, but especially the brain, may become affected. The partially nourished system of the child, cannot bear up against the disorders induced by dentition, and it falls a victim to hydrocephalus, or some other disease, against which, under a more natural mode of nurture, it might have successfully struggled. Nor does the mischief stop here, for, as the author truly observes, even supposing that the child escapes all the mischances of infancy, yet is its constitution, long after, and perhaps permanently, enfeebled, and it is always much more liable than the robust to the supervention of various congestive and inflammatory affections.

Dr. Davis especially deprecates the attempts, often made by many mothers, to bring up their children upon *arrow-root*, which they think supplies a nourishment equal to their own milk. Allowing it as an auxiliary to the mother's milk, during the first few weeks, he insists upon the impropriety of attempting to nourish a child entirely upon it, affording, as it does, such an inefficient degree of support.

Among other predisposing causes may be enumerated; certain peculiarities of temperament, and physical conditions of the brain, and the exanthematous and gastric fevers of infancy. An obstructed or inflammatory condition of the viscera of the cavities of the abdomen and chest predispose in like manner:

"First, by producing and sustaining an excess of vascular determination to the brain;—secondly, by exciting sympathetically a morbid action in the vascular tissues of the brain;—and, thirdly, by what I may be permitted to call a critical metastasis of the diseased actions of organs within the chest and abdomen to the vascular tissues of the encephalon."

Again, other causes are found, in long-continued disorders of the bowels, the abuse of opiates, the early use of stimulating food, and the excessive occupation of the mind, in which many children are engaged at schools. Diseases badly cured, or suffered to become chronic, and low inflammatory affections, occurring in the cachectic habit of body, often lay the foundation for hydrocephalus. Golis has observed the curious fact, that strong emotions on the part of the mother, when far advanced in pregnancy, predisposes the infant to disease of the brain; most of the children, who were born soon after the bombardment of Vienna in 1809, died convulsed in 10, 20, or 30 days—their brains exhibiting marks of inflammation, and the ventricles containing fluid.

### III. Exciting Causes.

Among these may be mentioned, concussion of the brain by violent movements of the body, as in rude sports, jolts of cradles, carriages, blows of the head, &c.; the too rapid suppression of discharges and cutaneous diseases; inflammations of the face, ears, mouth, &c.; want of cleanliness of the scalp; metastases of eruptive fevers. Dr. Davis believes, with the German writers, that many cases of hydrocephalus originate in *colds*, caught during the first few days after birth, which are also the causes of many cases of convulsions so frequent at that period of life. In reference to the assertion, of the occurrence of hydrocephalus as a consequence of vomiting, Dr. Davis thinks it unfounded; for, although he is much in the habit of employing emetics in acute diseases, he has never observed any such consequence to follow their use. The opinion, that suddenly suppressed diarrhœa often leads to the disease, he considers as well-founded, having met with several cases so originating. So, also, he has met with several examples of what Golis calls the tumultuous form of the disease, attributable to "bad management, and consequent sudden suppressions or retrocessions of the peccant fluids, incident to sundry discharges from large ulcers and extensive skin diseases."

### IV. Proximate Cause.

Very nearly half the work is occupied in discussing this point. We think the author has been most needlessly and injudiciously prolix: the fact, that hydrocephalus is itself a mere effect, and arises from a prior inflammatory state of the brain and its membranes, has now been often ably demonstrated, and is generally admitted; but supposing, for a moment, that the fact were less generally known than we believe it to be—we then think the author's time injudiciously employed in entering upon long criticisms of the erroneous ideas of Carmichael Smyth, and the imperfectly developed ones of Dr. Quin, not to mention a dissertation upon a case published in Venice in 1621. It would have been more to the purpose to have cited the opinions and experience of Drs. Clarke and Abercrombie, (both of whom treat of the disease as purely an inflammatory affection of the brain,) and some of the French writers. Strange to say, the names of no one of these authors is alluded to, and Dr. Davis appears to think, the demonstrating this view of the nature of the disease has never been efficiently performed by any one but Golis, and, that it even yet remains a disputed point. Any doubt which may remain, the author endeavours to dissipate, not by any original dissections, but by republishing a great number of the observations and dissections made by Golis, (whose work is well known in England through the medium of Dr. Gooch's translation) and some dissections by Dr. Cheyne. However this deficiency on his part is, we will admit, explained upon grounds truly satisfactory. "I have no inclination to multiply evidence in favour of the inflammatory origin of hydrocephalus, founded on results of cases which have from time to time occurred in my own practice, inasmuch as the majority of such opportunities are become simply matters of recollection to me, the post-mortem examinations of the last nine or ten years within my experience having been few and far between; the great majority of cases having yielded to the treatment adopted." 165.

### V. TREATMENT.

The treatment Dr. Davis has found so successful consists in free bleeding, the use of emetics, and the efficient application of cold.

1. *Bloodletting.*—The author complains, that great prejudice exists against the effectual employment of this means. "It accords with our universal experience, that whenever it becomes our duty to recommend the abstraction of blood from infants or very young children in a way to ensure the efficiency of the operation, the proposition is sure to be received with coolness in the first instance, if not eventually with repulsion or positive non-compliance." 229.

The best *time* for bleeding is the earliest possible period, after the nature of the disease has been positively decided upon. The inflammatory stage being preceded, often for some days, by premonitory symptoms, a question arises, whether the operation should be had recourse to during their existence, before the malady can be said to be fully established. Here is Dr. Davis' opinion :—

"I would answer, that during the pyrexia attendant upon the accession of an active fever, although its peculiar character might not be yet established, it could never be improper, but on the other hand a positive advantage, to have recourse to an abstraction of blood, upon a competent scale, to effect a subduction of the pyrexial actions of the arterial system....... All that could be required to justify the decision of advising the abstraction of blood under the supposed circumstances, would be a state of pyrexia as already assumed; and whether it might prove in the further progress of its development a case of small-pox, measles, acute hydrocephalus, or of any other fever whatever, the operation of a timely abstraction of blood would be a measure of unquestionable advantage in the calculation of its proving a disease of any pyrexial importance." 238.

This we think is going too far. When the case has arrived at the inflammatory stage, there can be no doubt of the necessity of free and immediate bleeding. As to the *quantity* to be abstracted, we again quote the author's words :

"I know but of one useful, safe, and satisfactory test; and that I beg to propose to the consideration of my reader, as the result of an anxious, cautious, and most extensive experience, during a period at least of about ten years. It is to carry the first bleeding, provided the opportunity is given to prescribe it at the onset of the disease, to full fainting. It is not a state of faintishness that is wanted; it is full and decided fainting. It is that state of enfeebled and reduced circulation, which, together with an occasional suspension of consciousness, is called deliquium animi. It is accompanied by an absence of all colour in the lips, and also in a great measure by a corresponding change of complexion of the whole countenance. The entire surface of the body, and especially of the forehead is suffused, during this condition of the circulation, with an excessive abundance of cold moisture." 241.

Dr. Davis' rule being to bleed until complete fainting occurs, the quantity taken is not of so much consequence: but, we subjoin his statement of the extent to which children may usually be bled, before fainting is produced:

| | | | |
|---|---|---|---|
| A child between one month and one year........ | 3 | to | 5 oz. |
| A child of .. .. .. two years ........ | 5½ | to | 6 oz. |
| ——— between three years and five years ........ | 6 | to | 10 oz. |
| ——— between six and ten years................ | 10 | to | 18 oz. |

At five years old and upwards, the blood may be taken, as usual, from the arm, but, at an earlier age, it is more conveniently abstracted by cupping-glasses, or by opening the jugular vein. If the bleeding be employed fully and promptly, it will seldom be necessary to repeat it; and it is far better and safer to attack the disease thus vigorously at first, than to employ smaller and successive bleedings. Much must depend upon the stage and degree of development of the disease. A greater loss of blood is usually required, in proportion to the degree of heat of the head. Although it is sometimes necessary to repeat the operation, this has rarely to be done more than once.

Of all *modes* of bleeding children, Dr. Davis thinks the application of the *cupping-glasses*, behind the ears, the best; but, as it requires considerable dexterity for its performance, it may often happen that a sufficiently adroit operator is not at hand. He suggests, that in towns and districts, where professed cuppers do not reside, the practitioners should select one of themselves, to whom all the cupping of the locality should be transferred by the rest. Where cupping cannot be performed, he recommends opening the *jugular vein*, but he is here again met with a difficulty. "In general practice I have found the younger part of our profession somewhat indisposed to perform this very simple operation. This indisposition has no doubt arisen from not having been taught to practise it under the eye and with the assistance of their senior and superior, during their apprenticeship." As a last resource only he would employ *leeches*: he thinks this a wretched mode of abstracting blood, as, from the slowness of the operation, a sufficient quantity of blood cannot be abstracted, in a moderately short time; and thus the general arterial action, upon which the continuance of the disease depends, cannot be subdued. This he considers explains the anomaly with regard to the practice of Golis, who, notwithstanding the correct view he took of the pathology of the disease, lost a very large proportion of his cases: had he employed cupping, in the place of leeches, the result might have been very different.

2. *Emetics.*—Dr. Davis places great reliance upon these. "Having secured as much control over the morbid arterial excitement of an incipient case of acute hydrocephalus as one sufficient abstraction of blood can be expected to give, the first important measure to be adopted immediately afterwards, with the view of maintaining, or even of extending our control over the action of the heart and arteries, should be to exhibit to the little patient, as soon as possible after his recovery from the fainting consequent upon the loss of blood, an active emetic. For a child of six months old, the proper dose should be a fourth or a fifth of a grain of tartarized antimony and about five grains of powered ipecacuanha. This form and quality of an emetic will rarely disappoint the expectations of the practitioner; whereas the pleasant draughts and mixtures made with the diluted wines of tartarized antimony and ipecacuanha, usually prescribed as emetics for young children, are occasionally chargeable with that result." 257.

This emetic will probably induce great vomiting and may even harass the patient for several hours; but then there is all the more probability, that the general arterial action will continue subdued. To ensure its action, fluids should, if necessary, be forced upon the child, but it will usually take the breast readily. Dr. Davis prefers the full action of an emetic, even should it be necessary to repeat it in a few hours, to inducing the horrible suffering attendant upon mere nausea.

3. *Cold to the Head.*—To enable us to examine the head properly, and to apply the requisite means, all the hair should be closely shaved off. The speedy reduction of its exalted temperature is of high importance. Sometimes the heat is greatest at the occiput, sometimes at other parts, usually at the forehead. We may at once ascertain its degree and situation, by applying both our hands for a few seconds to opposite parts of the head. The best means of reducing the heat is by the use of *Mackintosh's Water-cushion.*

"The mode of using this very convenient implement is to charge a cushion of this description to the extent of its entire capacity with the intended fluid, and immediately to allow one half of the whole fluid contained in the cushion to escape; and then suddenly to stop and secure the valve so as perfectly to prevent the escape of any more of the cold fluid, and at the same time to bar all intrusion of atmospheric air. The cushion will then contain half its capacity of an intensely cold fluid, and no air. The little patient's head is to be laid with its occiput and nape of the neck to repose on the middle of the cushion; which will have the effect of rapidly reducing the excessive heat of the head." p. 266.

The fluid will require changing within the half-hour. As long as the heat of the head continues, the child testifies great pleasure on the re-application of the cold; and when, from a diminution of the temperature having taken place, it manifests alarm at the renewal, it should be suspended or discontinued.

4. *Blisters.*—These, Dr. Davis considers as of very little utility, and, justly observes, that they will prove positively hurtful, when applied prior to the use of depletion. He prefers large ones which cover a considerable space of the parietal region on both sides; thus leaving the forehead free for the application of evaporating lotions, and the occiput at liberty to repose on the cushion.

5. *Mercury.*—"Mercury, it is well known, is a medicine of no little importance in the treatment of inflammatory diseases. As a remedy for acute hydrocephalus it has been recommended as a cholagogue, a purgative, and as a power capable of repressing inflammatory action. As a *purgative,* I am in the habit of recommending a combination of two or three grains of calomel, and sometimes twice that quantity, with six, eight, or twelve grains of jalap, according to the age of the patient, as soon as the sickness shall have ceased after the operation of the emetic. To prevent uneasiness from griping I seldom omit adding a few grains of ginger. In cases of known obstinate constipation I have sometimes considered it useful to add a quarter or half a drop, or even more, when authorized by the age of the patient, of croton oil." p. 271.

When using it as a *cholagogue,* in the suspended and disordered states of the functions of the liver, which so often precede or accompany hydrocephalus, Dr. Davis recommends from one to three grains of calomel, to be given uncombined every three hours.

In regard to producing the *sialogogue* effects of mercury, the author thus expresses himself, after adverting to the bad effects, which sometimes result from too free salivation, and the necessity of caution in the administration of the larger doses of this mineral.

"The few cases of recovery from acute hydrocephalus which we find recorded as having taken place in the absence of early bleeding, are, I think, principally, if not exclusively, to be attributed to this constitutional action of calomel as a resolvent of inflammatory action. But this power is rarely exerted to the extent of saving life. I never, myself, depend upon it; and, indeed, I seldom use it at all, excepting as an auxiliary to the lancet, or as an almost hopeless substitute for its use, when considered too late to have recourse to adequate repeated abstractions of blood with any considerable hopes of a successful issue." p. 275.

Dr. Davis concludes his work by recapitulating the principal premonitory symptoms of hydrocephalus, on the appearance of which parents should lose no time in applying for medical aid. He also presents us with abstracts of twelve cases, which terminated fatally, for want of timely or appropriate treatment; and the details of six others, which have been very recently successfully treated, at the University College Hospital.

The author repeatedly characterises his book as "this little volume," "this little work": we think 300 pages octavo ample room for treating the subject of acute hydrocephalus, and that the work even might have been reduced in size with advantage. The first part, demonstrating the pathology of hydrocephalus, is quite uncalled for, as adding nothing to what is well known upon the subject. The latter portion we receive thankfully. Not that the doctrines advocated in it are by any means new, as Dr. Clarke, more than twenty years ago advocated cupping to faintness and opening the jugular vein, while Dr. Maxwell of Dumfries, as quoted by Dr. Joy, in the Cyclopædia of Practical medicine, cured sixty out of ninety patients by bleeding from the jugular vein until fainting supervened. Still, it is encouraging to find a physician of Dr. Davis's experience, speaking so confidently of the frequent curability of a disease so terrible as hydrocephalus; and we do not doubt, that the publication of his opinion will be the means of exciting renewed attention to the question of early and free depletion. It must not however be forgotten, that the system of indiscriminate bleeding in head affections, which, for a while, followed the publication of Dr. Clarke's work on Diseases of Children, has in our own day found few admirers. The reducing a child to a state of perfect syncope, when merely general febrile action is present, should not be rashly undertaken, and when put into operation before decided symptoms develop themselves, will often have been unnecessary. Doubtless many cases so treated and recovering would never have turned out to be cases of hydrocephalus at all. These cautions are the more necessary, as, although the author forcibly shews the good effects which may result from extensive bleeding, he never, in the remotest manner, hints at the possibility of over-depletion occurring, nor does he indicate those obscure and difficult cases of head-disease, in which depletion of any kind would be injurious or even destructive.

